# AMPK-dependent and independent effects of AICAR and compound C on T-cell responses

**DOI:** 10.18632/oncotarget.9277

**Published:** 2016-05-10

**Authors:** Enyu Rao, Yuwen Zhang, Qiang Li, Jiaqing Hao, Nejat K. Egilmez, Jill Suttles, Bing Li

**Affiliations:** ^1^ Department of Microbiology and Immunology, University of Louisville, Louisville, KY, USA; ^2^ Department of Burn and Plastic Surgery, Shandong Provincial Hospital Affiliated to Shandong, University, Jinan, Shandong, China

**Keywords:** AMPK, T cell responses, Immunology and Microbiology Section, Immune response, Immunity

## Abstract

As a master metabolic sensor, AMP-activated protein kinase (AMPK) is involved in different fundamental cellular processes. Regulation of AMPK activity either by agonists (*e.g.*, AICAR) or by antagonists (*e.g.*, Compound C) has been widely employed to study the physiological functions of AMPK. However, mounting evidence indicates AMPK-independent effects for these chemicals and how they regulate immune cell functions remains largely unknown. Herein, using T cells from AMPK conditional knockout mice and their wild type littermates, we demonstrate that AICAR and Compound C can, indeed, activate or inhibit AMPK activity in T cells, respectively. Specifically, AICAR inhibits, but Compound C promotes, Ca^2+^-induced T cell death in an AMPK-dependent manner. In contrast, our data also demonstrate that AICAR and Compound C inhibit T cell activation and cytokine production in an AMPK-independent manner. Moreover, we find that the AMPK-independent activity of AICAR and Compound Cis mediated via the mTOR signaling pathway in activated T cells. Our results not only reveal the critical role of AMPK in regulating T cell survival and function, but also demonstrate AMPK-dependent and independent rolesof AICAR/Compound C in regulating T cell responses, thus suggesting a context-dependent effect of these “AMPK regulators”.

## INTRODUCTION

As an evolutionarily conserved metabolic sensor, AMPK has been shown to regulate various aspects of cellular fundamental functions including cell proliferation, survival and metabolism [1-3]. The AMPK complex is composed of three subunits, one catalytic α subunit and two regulatory β and γ subunits. In different tissues of mammals, AMPK displays distinct expression pattern of subunits, which contain two α subunits (α1, α2), twoβ subunits (β1, β2), and three γ subunits (γ1, γ2 and γ3) [2, 4]. AMPK activity can be regulated by intracellular factors, such as the cellular AMP/ATP ratio, as well as auto-inhibitory features and phosphorylation status of its subunits. Full activation of AMPK requires specific phosphorylation of the α subunit at the conserved threonine residue (Thr172) by upstream kinases including LKB1, CAMKKs and TAK1. Protein phosphatases 2A and 2C also regulate the activation of AMPK by dephosphorylation of Thr172 [1, 5]. It is well-established that activation of AMPK is critical in restoring the intracellular energy balance to sustain cell survival and function under stress via turning off ATP-consuming anabolic pathways and stimulating ATP-producing catabolic processes [2, 4, 6].

Chemical reagents that target AMPK activity have been widely used to investigate cellular functions of AMPK [7-10]. For example, AICAR (5-Aminoimidazole-4-carboxamide ribonucleoside) is the first identified AMPK agonist, which is commonly used to activate AMPK in many *in vitro* and *in vivo* studies [11, 12]. Mechanistically, AICAR is converted intracellularly to ZMP, an intermediate in the late steps of *de novo* purine biosynthesis, which mimics AMP and activates AMPK regardless of cellular energy status [11, 13]. AICAR is being used clinically to protect against cardiac ischemic injury and to improve myocardial protection in coronary artery bypass grafting [14-16]. Moreover, AICAR is also a promising drug for the treatment of B-cell neoplasms and chronic lymphocytic leukemia [17-19]. In contrast, Compound C (6-[4-(2-Piperidin-1-ylethoxy) phenyl]-3-pyridin-4-ylpyrazolo [1,5-a]pyrimidine) (CC), is well-known for its potent inhibitory effect on AMPK activation. In combination with AMPK agonists (e.g. AICAR), Compound C is often used as an AMPK antagonist to study AMPK-dependent cellular events [5, 20, 21]. However, mounting evidence indicates AICAR and Compound C are able to regulate cellular functions *via* AMPK-independent mechanisms [19, 22-30]. In addition, Compound C has been shown to inhibit activities of many other kinases, such as ERK8, ALK2, Src, Lck, *etc*, besides AMPK [31, 32]. Thus, pharmacological application of AICAR or Compound C may result in both AMPK-dependent and independent effects in different types of cells.

In our previous studies using AMPK conditional knockout mice, we have demonstrated that expression of AMPK in T cells is indispensable for their activation, but critical to promoting their survival and anti-tumor functions in mouse tumor models [10]. Thus, clinical application of AMPK agonists/antagonists will most likely influence T cell survival and function. Although the effects of AICAR and Compound C on T cell activity has been studied [30, 33-37], whether these effects are dependent or independent of AMPK is still unclear. Here, using AMPK-deficient T cells, we investigated the effects of AICAR and Compound C on T cell survival and function. We found that AICAR promoted, but Compound C inhibited, Ca^2+^ signaling-induced T cell death in an AMPK-dependent manner. Importantly, both AICAR and Compound C exerted an inhibitory effect on T cell activation and function in an AMPK-independent manner.

## RESULTS

### AICAR promotes, but Compound C inhibits, AMPK activation in T cells

**Figure 1:**
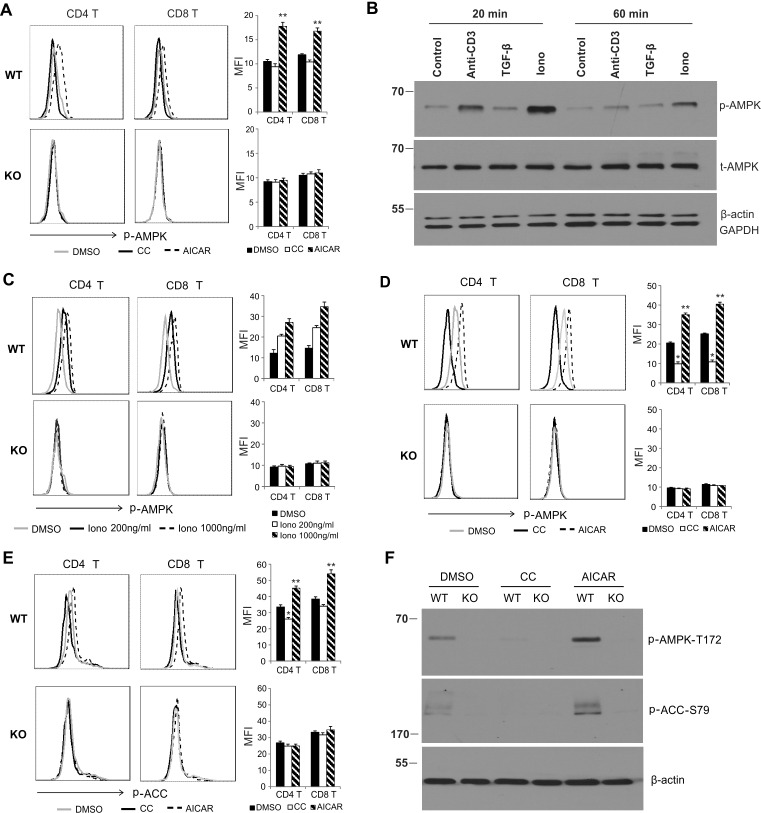
AICAR promotes, but Compound C inhibits, AMPK activation in T cells **A.** Cells from lymph nodes of WT and KO mice were treated with DMSO, Compound C (CC, 10μΜ) or AICAR (500μM) for 30 minutes and were analyzed for p-AMPK^T172^ levels in CD4^+^ and CD8^+^ T cellsby intracellular staining. The mean value of median fluorescence intensity (MFI) in DMSO, CC or AICAR group is shown in the right panel (**, *p* < 0.01 as compared to DMSO group). **B.** LN cells were treated with anti-CD3 (5μg/ml), TGF-β (5ng/ml) or ionomycin (1μg/ml), respectively. Cells were collected for western blot analysis at indicated time points. **C.** LN cells were treated with DMSO or indicated concentrations of ionomycin (200ng/ml or 1000ng/ml) for 20 minutes. p-AMPK^T172^ levels in CD4^+^ and CD8^+^ T cells were analyzed by intracellular staining. (D, E) Cells from lymph nodes of WT and KO mice were pretreated with DMSO, AICAR (500μM) or CC(10μM) for 30 minutes and then stimulated with PMA/Ionomycin (P/I) for another 20 minutes, p-AMPK^T172^ **D.** and p-ACC^S79^ **E.** in CD4^+^ and CD8^+^ T cells were analyzed by intracellular staining. MFI in DMSO, CC or AICAR-treated group is shown in the right panel (*, *p* < 0.05; **, *p* < 0.01 as compared to DMSO group). **F.** Sorted CD4^+^ T cells were pretreated with DMSO, CCand AICAR for 30 minutes and then followed by Ionomycinstimulation for another 20 minutes. Cells were collected for analysis of p-AMPK^T172^ and p-ACC^S79^ by western blotting.β-Actin was used as the loading control. Data represent one of at least three independent experiments.

Although AICAR/Compound C have been commonly used as an agonist/antagonist of AMPK, respectively, whether or not they are able to activate/inhibit AMPK in T cells remains unclear [18, 30, 34]. Our previous data demonstrated that AMPK is specifically deleted in T cells from CD4-Cre^+^ AMPKα1*fl/fl* (KO) mice, but is intact in T cells from CD4-Cre- AMPKα1*fl/fl* (WT) mice [10]. We thus continued to use this model to dissect the effects of AICAR/Compound C on AMPK in T cells. We first measured the AMPK activation using resting T cells from lymph nodes of WT and KO mice. Intracellular staining of phosphorylation of AMPK Thr-172 (p-AMPK) showed that AMPK was not or only weakly activated in resting WT T cells as compared to KO T cells. Interestingly, treatment with AICAR significantly increased phosphorylation of AMPK in WT T cells, but not in KO T cells, suggesting a specific activation of AMPK with AICAR. We did not observe any obvious inhibition of p-AMPK with Compound C treatment (Figure 1A), which may be due to the non- or weak activation of AMPK in resting T cells. As Ionomycin (Iono) was able to induce much stronger AMPK activation than anti-CD3 antibody or TGF-β in LN cells (Figure 1B), and it increased the levels of p-AMPK in WT T cells in a dose-dependent manner (Figure 1C), we next measured the effects of AICAR/Compound C on AMPK activation using Iono-activated T cells. Importantly, pretreatment of T cells with AICAR enhanced, but Compound C suppressed, phosphorylation of AMPK in Iono-activated T cells from WT mice, but not from KO mice, further suggesting a specific effect of AICAR and Compound C on AMPK activity in activated T cells (Figure 1D). We also investigated the impact of AICAR/Compound C treatment on acetyl-CoA carboxylase (ACC), the downstream target of activated AMPK in T cells. Similarly, AICAR promoted, while Compound C inhibited, phosphorylation of ACC (Ser-79) in Iono-activated CD4^+^ and CD8^+^ T cells from WT mice (Figure 1E). Using Western blot analysis, we further confirmed that AICAR enhanced, but Compound C inhibited, the phosphorylation of AMPK and ACC in T cells from WT mice, but not from KO mice (Figure 1F). Altogether, using CD4-Cre-AMPKα1*fl/fl* mice, our data clearly indicate a specific AMPK activation/inhibition effect of AICAR/Compound C in T cells.

### AICAR inhibits, but Compound C promotes, Ca^2+^-induced T cell death in an AMPK-dependent manner

**Figure 2:**
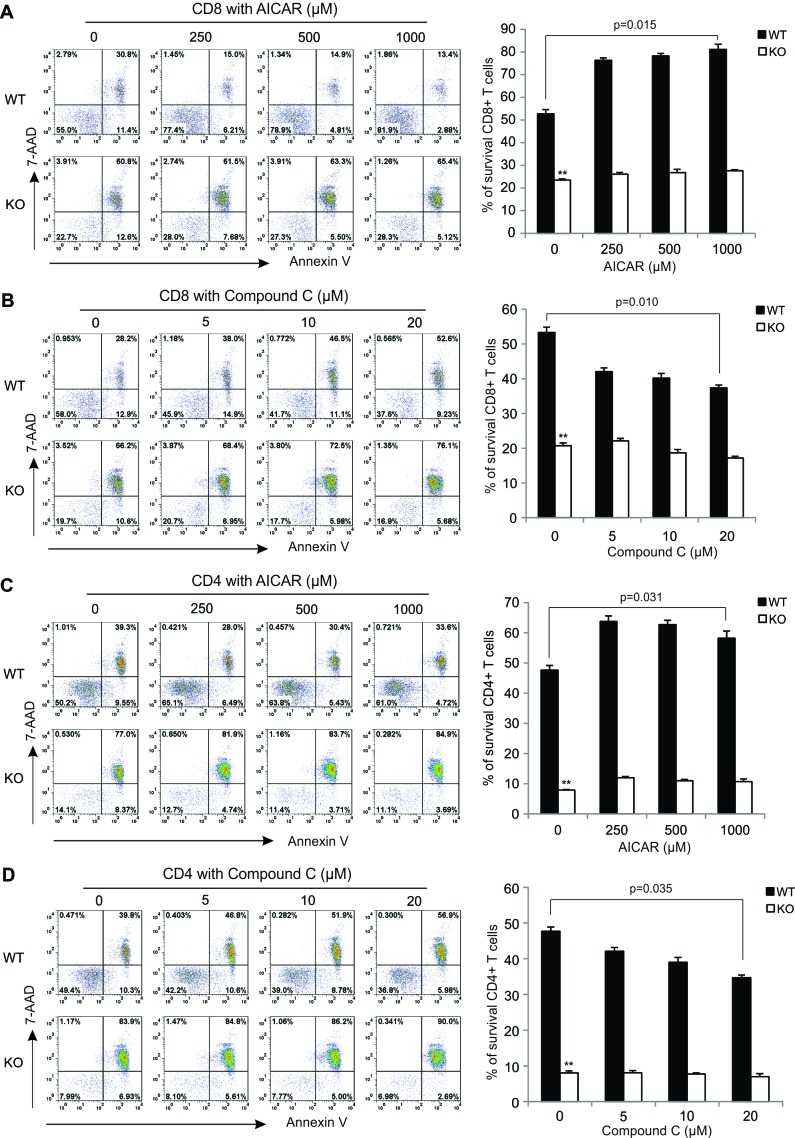
AICAR inhibits, but Compound C promotes, Ca^2+^ signaling-induced T cell death in an AMPK-dependent manner Cells from lymph nodesof WT and KO mice were pretreated with indicated concentrations of AICAR or Compound C for 30 minutes, and then stimulated with PMA(10ng/ml)/Ionomycin (1000ng/ml) for 6 hours. The survival of CD8^+^ T cells and CD4^+^ Tcells was analyzed with Annexin V and 7-AAD staining. The representative dot blots showing the survival of CD8^+^ T cells treated with AICAR **A.** or Compound C **B.**,and the survival of CD4^+^ T cells treated with AICAR **C.** and Compound C **D.** are shown in theleft panels. The right panels are the average of the survived cells shown as mean ± SD (**, *p* < 0.01 as compared to WT group).

Calcium signals are essential to the cell functions. Intracellular calcium overloading or perturbation could trigger cell death [40]. The dysregulated Ca^2+^ responses are also associated with various pathophysiological processes in several autoimmune and inflammatory diseases [41]. In our previous studies, we found AMPK activation protects T cells against high concentration Ionomycin (Ca^2+^ ionophore)-induced cell death [10] and PMA treatment has no obvious effects on T cell survival (Figure S1). We wondered whether treatment with AICAR/Compound C affects Ca^2+^-induced T cell death through regulation of AMPK activation. To this end, we pretreated cells from lymph nodes with different concentrations of AICAR or Compound C before PMA/Ionomycin (1000ng/ml) treatment, and measured T cell survival at different time points via staining of 7-AAD and Annexin V. In agreement with our previous observations, AMPK activation increased the survival of CD8^+^ T cells in WT mice (57.6%) as compared to those in KO mice (23.3%) under PMA/Ionomycin stimulation for 6 hours. Interestingly, pretreatment with AICAR further promoted the survival of WT CD8^+^ T cells, but not in CD8^+^ T cells from KO mice (Figure 2A). In contrast, pretreatment with Compound C significantly decreased the survival of WT CD8^+^ T cells, but not KO CD8^+^ T cells (Figure 2B). These data suggest an AMPK-specific effect of AICAR and Compound C on CD8^+^ T cell survival. When we further analyzed the impact of AICAR/Compound C treatment on the survival of CD4^+^ T cells, we observed the same effects of AICAR/Compound C on the survival of CD4^+^ T cells as what we observed in CD8^+^ T cells (Figure 2C, 2D). In addition, we also measured T cell survival after longer period of activation with PMA/Ionomycin (12 hours) and confirmed the similar effects of AICAR and Compound C on T cell survival (Figure S2). Thus, all data suggest that AICAR promotes, but Compound C inhibits, Ca^2+^-induced T cell death in an AMPK-dependent manner.

### AICAR and Compound C suppress T cell activation in an AMPK-independent manner

**Figure 3:**
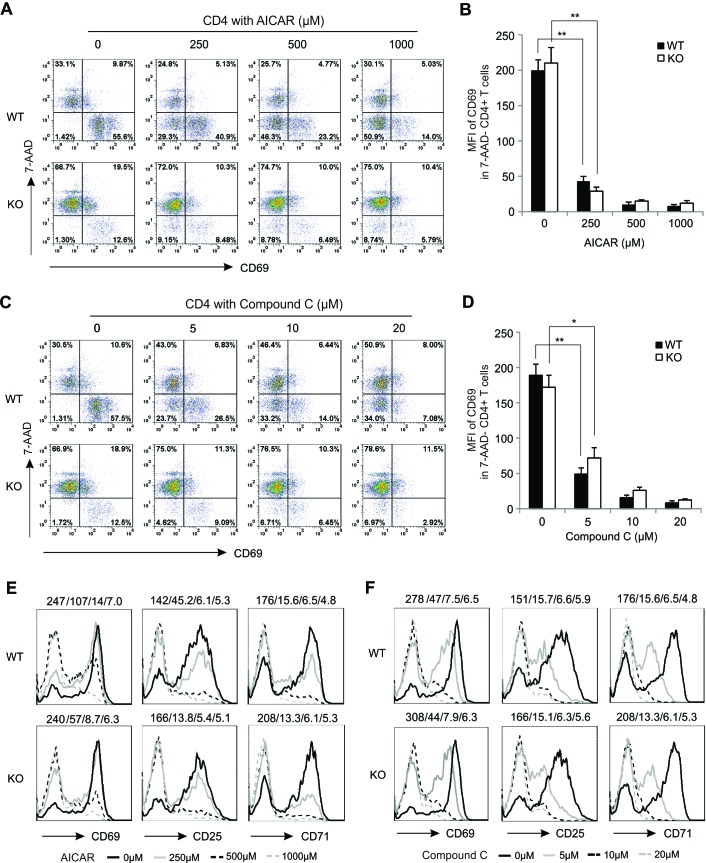
AICAR and Compound C suppress T cell activation in an AMPK-independent manner Cells from lymph nodes of WT and KO mice were pretreated with indicated concentrations of AICAR or Compound C for 30 minutes, and then stimulated with PMA(10ng/ml)/Ionomycin (1000ng/ml) for 6 hours. Expression of CD69 in CD4^+^ T cells was analyzed by flow cytometricstaining with anti-CD69 antibody and 7-AAD. **A.** Dot plot of anti-CD69 antibody and 7-AAD staining in CD4^+^ T cells with AICAR treatment. **C.** Dot plot of anti-CD69 antibody and 7-AAD staining in CD4^+^ T cells with Compound C treatment. The MFI of CD69 expression with AICAR**B.** or Compound C **D.** treatment in 7-AAD- CD4^+^ T cells. (E, F)Cells from lymph nodes of WT and KO mice were stimulated with coated anti-CD3 (5μg/ml) and anti-CD28(1μg/ml) antibodies for 24 hours after pretreatment with indicated concentrations of AICAR**E.** or Compound C**F.**. Expression ofCD69, CD25 and CD71 in CD4^+^ T cells was analyzed by flow cytometric staining. The MFI is shown above the histograms and represents0 μM, 250μM, 500μM and 1000μM of AICAR treatment in panel E, and 0 μM, 5 μM, 10 μM, 20μM of CC treatmentin panel F from left to right, respectively. Data represent one of at least three independent experiments(*, *p* < 0.05; **, *p* < 0.01).

After determining the effects of AICAR/Compound C on T cell survival, we next investigated whether these agents affect T cell activation. The early T cell activation marker CD69 was expressed at low levels on resting lymphocytes (Figure S3), but was rapidly upregulated following activation. Interestingly, AICAR inhibited CD69 expression on live CD4^+^ T cells (7-AAD-) in a dose-dependent manner in both WT and KO mice (Figure 3A). Moreover, CD69 was expressed at similar levels on live T cells from WT and KO mice with or without AICAR treatment, implying that AMPK deficiency has no impact on CD69 expression (Figure 3B). The low percentage of 7-ADD^−^ CD69^+^ T cells in KO mice may be due to the elevated cell death in the absence of AMPK. Thus, AICAR inhibited CD69 expression regardless of AMPK expression. Unexpectedly, treatment of Compound C also inhibited CD69 expression on PMA/Ionomycin-activated CD4^+^ T cells in the same pattern as AICAR treatment (Figure 3C, 3D). To further confirm these observations, we measured CD69 expression on T cells activated with anti-CD3/CD28 antibodies. Both AICAR and Compound C inhibited CD69 expression on anti-CD3/CD28-activated T cells from WT and KO mice. In addition, other T cell activation markers, including CD25, CD71, were also inhibited by AICAR or Compound C treatment (Figure 3E, 3F). Taken together, these data suggest that T cell activation does not require AMPK expression and both AICAR and Compound C are able to inhibit T cell activation in an AMPK-independent manner.

### AICAR and Compound C inhibit T cell cytokine production in an AMPK-independent manner

**Figure 4:**
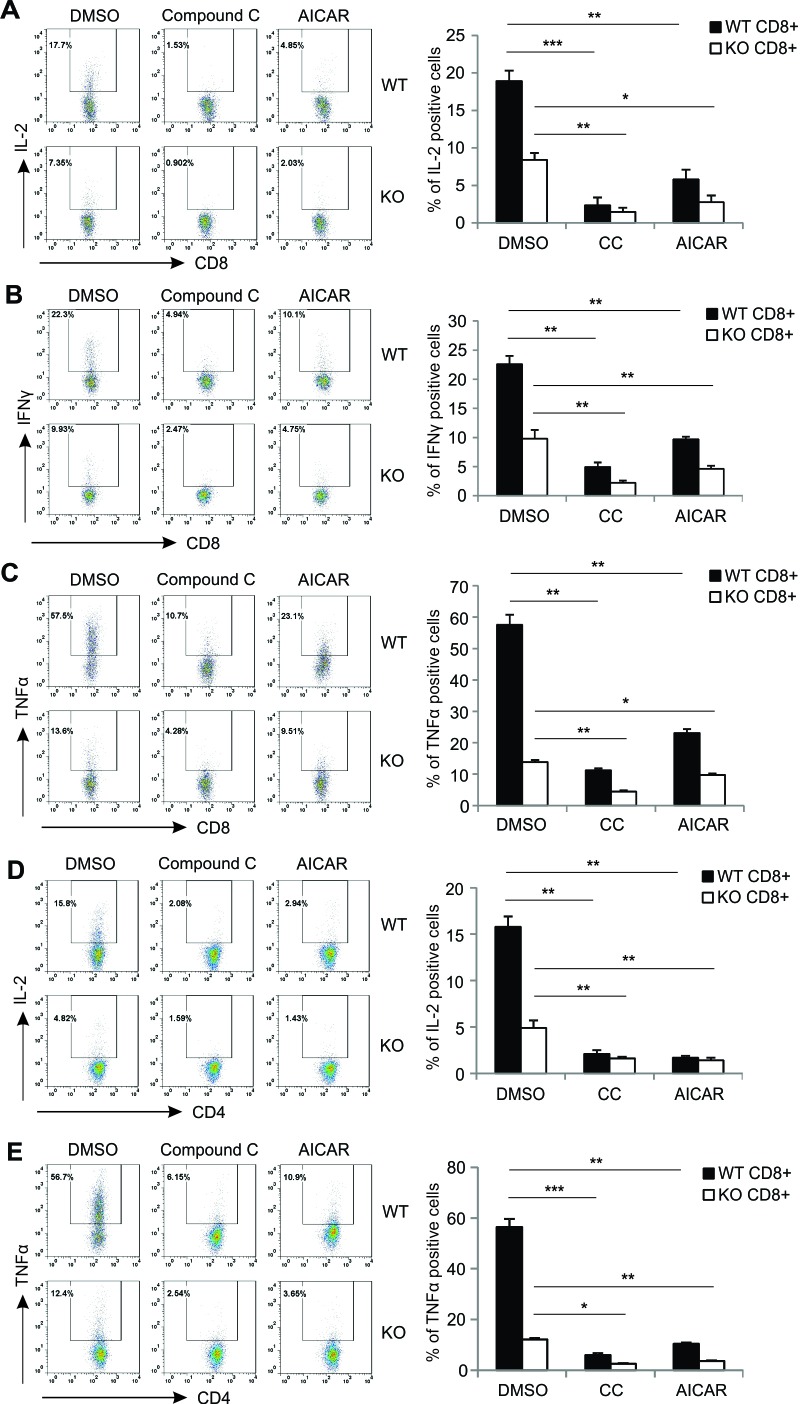
AICAR and Compound C inhibit cytokine production in PMA/Ionomycin-activated T cells Cells from lymph nodes of WT and KO mice were pretreated with DMSO, Compound C (10μM) or AICAR (500μM) for 30 minutes, and then stimulated with PMA(10ng/ml)/Ionomycin (1000ng/ml) and Golgiplug for 5 hours. Cells were collected and stained for cytokines production. **A.** IL-2 production in CD8^+^ T cells. **B.** IFNγ production in CD8^+^ T cells.**C.** TNFα production in CD8^+^ T cells. **D.** IL-2 production in CD4^+^ T cells. **E.** TNFα production in CD4^+^T cells. The mean values and SD of each cytokine are shown in the right panel(*, *p* < 0.05; **, *p* < 0.01; ***, *p* < 0.001).

**Figure 5:**
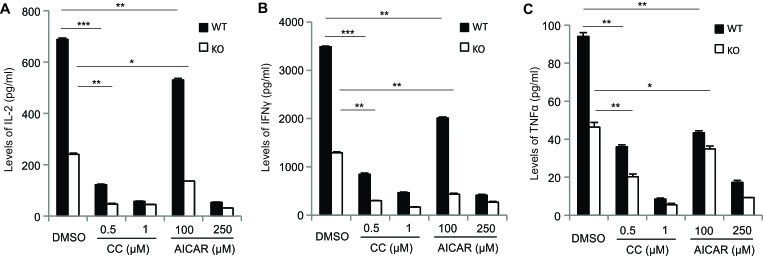
AICAR and Compound C inhibit cytokines in anti-CD3/CD28 activated T cells Cells from lymph nodes of WT and KO mice were cultured with coated anti-CD3 antibody (5ug/ml)/anti-CD28 antibody (1ug/ml) and indicated concentrations of AICAR or Compound C for 24 hours. Supernatants were collected for measurement of IL-2 **A.**, IFNγ **B.** and TNFα **C.** by ELISA (*, *p* < 0.05; **, *p* < 0.01; ***, *p* < 0.001).

We next determined whether AICAR or Compound C affects T cell function by measurement of cytokine production in activated T cells. In line with our previous data [10], deletion of AMPK in T cells greatly impaired production of IL-2, IFNγ and TNFα in PMA/Ionomycin-activated CD8^+^ T cells (DMSO control group in Figure 4A, 4B, 4C), implying that AMPK activation contributes to cytokine production in T cells. Interestingly, when we pretreated T cells with either AICAR or Compound C before PMA/Ionomycin activation, the production of cytokines, including IL-2, IFNγ and TNFα, were inhibited substantially in CD8^+^ T cells from both WT and KO mice (Figure 4A, 4B, 4C), suggesting that AICAR- or Compound C-mediated cytokine inhibition is independent of AMPK expression/activation in CD8^+^ T cells. Of note, similar inhibitory effects were also observed in CD4^+^ T cells, regardless of AMPK expression (Figure 4D, 4E). As AMPK deficiency promoted Ca^2+^ overload-induced T cell death (*e.g*. 1000ng/ml Ionomycin), which may contribute to the reduced cytokine production in KO T cells, we further measured cytokine production in T cells stimulated with a low dose of Ionomycin (200ng/ml), which induced no discernible T cell death in the presence or absence of AICAR or Compound C between WT and KO mice (Figure S4). We found that AMPK expression was still able to promote cytokine production in both CD4^+^ and CD8^+^ T cells (left panel, Figure S5). However, treatment with either AICAR or Compound C inhibited cytokine production irrespective of AMPK expression in T cells (middle and right panels, Figure S5). Furthermore, we measured cytokine production in supernatants collected from anti-CD3/CD28-activated T cells and demonstrated that both Compound C and AICAR inhibited levels of IL-2, IFNγ and TNFα regardless of AMPK expression (Figure 5). Thus, our data indicate that, although AMPK is critical in promoting cytokine production in Ca^2+^- and TCR-activated T cells, the cytokine-inhibitory effects of AICAR/Compound C on T cells are independent of AMPK.

### Treatment of AICAR and Compound C inhibits the mTOR signaling pathway in T cells

**Figure 6:**
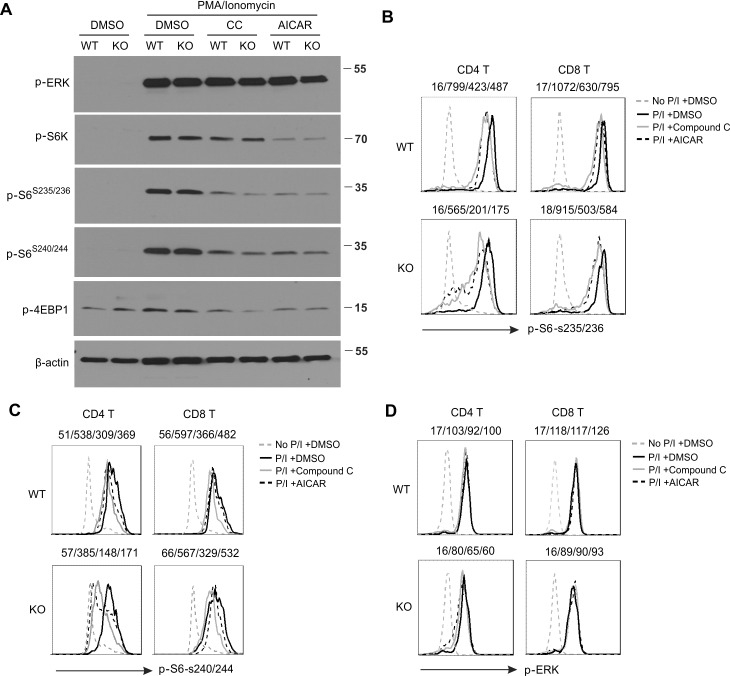
Treatment of AICAR and Compound C inhibits mTOR signaling in T cells CD4^+^ T cells separated from lymph nodes of WT and KO mice with a flow sorter were pretreated with DMSO, Compound C (10μM) and AICAR (500μM) for 30 minutes, and then stimulated with PMA(10ng/ml)/Ionomycin (1000ng/ml) for 20 minutes. Phosphorylation of ERK, S6K, S6P, 4EBP1 in total cells was analyzed by western blotting **A.**. Intracellular staining was performed to analyze the phosphorylation of p-S6^S235/236^ **B.**, p-S6^S240/244^ **C.** and p-ERK^T202/Y204^ **D.** in individual populations of CD4^+^ and CD8^+^ T cells. The values of MFI are shown above the histograms and representtreatments of no P/I control, P/I+DMSO, P/I+CC and P/I+AICAR from left to right, respectively. Data represent one of at least two independent experiments.

To dissect the possible mechanisms of AMPK-independent effects of AICAR and Compound C in T cells, we measured several major signaling pathways related to T cell function, including ERK, S6K, S6P, 4EBP1 in the presence or absence of AICAR/Compound C. As shown in Figure 6A, neither AICAR nor Compound C affected ERK phosphorylation in activated T cells. Considering the inhibitory effect of AICAR and Compound C on T cell function, it is unlikely that ERK signaling is the major pathway involved in the AICAR/Compound C-mediated effects. Interestingly, phosphorylation of S6K, S6P and 4EBP1 was greatly inhibited by either AICAR or Compound C treatment in T cells, suggesting a critical role of mTOR in AICAR/Compound C-mediated effects. By taking advantage of the single cell intracellular staining techniques, we demonstrated that treatment with either AICAR or Compound C reduced phosphorylation of Serine 235/236 of S6P (p-S6^S235/236^) in both CD4^+^ and CD8^+^ T cells (Figure 6B). As S6P can be phosphorylated on other sites, such as Serine 240/244, we also measured the effect of AICAR and Compound C on the phosphorylation of Serine240/244 (p-S6^S240/244^), and found the same inhibitory effect (Figure 6C). In agreement with our Western blotting results, AICAR and Compound C exhibited no obvious effects on the phosphorylation of ERK in T cells from both WT and KO mice (Figure 6D). Taken together, our data suggest that AICAR and Compound C may inhibit T cell activation and function via suppression of mTOR pathway.

## DISCUSSION

Due to the critical role of AMPK in the regulation of fundamental cellular functions, pharmacological modulation of AMPK activity has been widely used in experimental and clinical studies. Targeting AMPK by different agonists, such as AICAR and metformin, has been shown to exhibit therapeutic benefits in different diseases, including type II diabetes, cardiac ischemic injury and tumor development [7, 8]. While much attention has been focused on how modulation of AMPK activity affects non-hematopoietic cell pathology, the effect of AMPK agonists/antagonists on immune cell survival and function is yet to be determined. Using AMPK conditional knockout mice, we and others have previously demonstrated that AMPK is dispensable for T cell development and proliferation in normal physiological conditions, but indispensable for T cell survival and anti-tumor functions under tumor or other metabolic stresses [10, 42, 43]. In this follow-up study, we further investigated the effects of AICAR/Compound C treatment on T cell responses. We found that treatment with AICAR or Compound C results in pleiotropic effects, i.e. an AMPK-dependent effect on T cell survival and an AMPK-independent activity on T cell activation and function.

AICAR/Compound C is commonly used as an agonist/antagonist to study AMPK-dependent cellular pathways. By specific deletion of AMPK in T cells using CD4-Cre-AMPK *fl/fl* mice, we confirmed that AICAR and Compound C can indeed activate or inhibit AMPK, respectively, in T cells from WT mice (Figure 1). Importantly, treatment with AICAR or Compound C has no impact on AMPK KO T cell death, but respectively promotes or inhibits the survival of AMPK WT T cells in response to high concentration Ionomycin-activated T cells death (Figure 2). These data further substantiate the pro-survival role of AMPK in T cells, and reveal an AMPK-dependent effect of AICAR/Compound C on T cell survival. It is worth noting that the AMPK-dependent effect on T cell survival is not prominent when T cells are activated with anti-CD3/CD28 signals, which may be attributed to the weak activation of AMPK under the anti-CD3 stimulation (Figure 1B).

During our previous studies, we also noticed that AMPK deficiency has no influence on the expression of T cell activation markers, such as CD25, CD69, *etc*., implying that AMPK is dispensable for T cell activation. In the present study, we found that these early activation markers are only expressed in 7-AAD^−^ live T cells after activation regardless of AMPK expression. Although the overall expression levels of these markers are higher in total T cells from WT mice than from KO mice, the MFI of these markers on live T cells is comparable in both strains (Figure 3). Thus, AMPK deficiency seems to impact cell survival, but not activation, of T cells. More interestingly, both AICAR and Compound C significantly inhibit T cell activation as determined by the decreased expression of activation markers. Thus, these results further indicate that AMPK is not involved in T cell activation. In line with other studies that reported AMPK-independent activity of AICAR/Compound on cellular physiology [19, 24-27, 44], our data provide new evidence that these reagents inhibit T cell activation in an AMPK-independent manner.

In addition, AMPK-deficient T cells exhibit reduced cytokine production as compared to AMPK-sufficient controls (Figure 4). This could be explained by two possibilities: 1) AMPK deficiency may promote elevated cell death during T cell activation, thereby leading to reduced cytokine production. 2) AMPK signaling itself may regulate cytokine production pathways. To address this question, we activated T cells either with overload Ca^2+^ signals which mainly induce T cell death, or with low Ca^2+^ signals which mainly induce T activation, and measured intracellular cytokine production under both conditions (Figure 4 and Figure S5). Our results suggest that AMPK affects T cell cytokine production via both pathways. Intriguingly, addition of either AICAR or Compound C significantly suppresses cytokine production in PMA/Ionomycin-activated T cells from both WT and KO T cells. Moreover, similar results were observed when T cells were stimulated with anti-CD3/CD28 signaling (Figure 5). Of note, the cytokine inhibitory effects of AICAR/Compound C are not due to the non-specific toxicity of these compounds as none of the doses used induced cell death in our experimental settings (Figure 2, Figure S3). Thus, these data provide additional evidence that AICAR/Compound C inhibits T cell function independent of AMPK status. It has been shown that AICAR and Compound C can regulate T cell function and promote T cell anergy by inhibiting different pathways, such as PKC, NFAT, AP1 and GSK-3β [33-36]. During the investigation of possible mechanisms by which AICAR/Compound C induces AMPK-independent inhibition of T cell activation and function, our results suggest that they may also exert inhibitory effects through suppressing mTOR activation in activated T cells (Figure 6).

So far, the exact role of AMPK in T cell function is not fully understood and sometimes controversial. Depending on the experimental setting, different studies demonstrated that AMPK is either dispensable, or can influence IFNγ production in T cells [33-35, 45-47]. While it is known that AMPK is dispensable for T cell development, AMPK deficiency has been shown to impair the generation of memory T cells [45]. By specific deletion of AMPK in T cells using a genetic approach, our studies reveal several important functions of AMPK in T cells. 1) AMPK protects T cells against Ca^2+^-induced cell death. 2) AMPK promotes T cell function by enhancing cytokine production. 3) AICAR and Compound C exert both AMPK-dependent and independent effects in T cells depending on different functional context. In the context of T cell early activation and cytokine production, both AICAR and Compound C inhibit these events independent of AMPK. However, in the setting of Ca^2+^-induced T cell activation and death, AICAR and Compound C can either promote or inhibit T cell survival in an AMPK-dependent manner. Taken together, our data clarify the role of AMPK in T cells, as well as shed light on the implications of chemical intervention of AMPK activity in different diseases *in vivo.*

## MATERIALS AND METHODS

### Mice

Floxed *Prkαα1* mice were purchased from Jackson Laboratory and CD4-Cre mice were from purchased from Taconic. Floxed *Prkαα1* mice were crossed to CD4-cre mice to conditionally delete AMPKα1 expression in T cells during the double positive stage of T cell development. Genotyping of mice was performed by PCR with following primers: CCT GGA AAA TGC TTC TGT CCG TTT G and ACG AAC CTG GTC GAA ATC AGT GCG for Cre, CCC ACC ATC ACT CCA TCT CT and AGC CTG CTT GGC ACA CTT AT for floxed *Prkaα1*. Eight to twelve-week-old AMPKα1*fl/fl* CD4-Cre^+^ (KO) mice and AMPKa1*fl/fl* CD4-Cre^−^ (WT) littermates were used in experiments. All mice were bred and maintained in the animal facility in accordance with protocols approved by the Institutional Animal Care and Use Committee.

### Cell culture and treatment

Cells were prepared from lymph nodes (LN cells) and cultured with RPMI 1640 with L-glutamine (Corning Cellgro), 5% FBS and 50μg/ml gentamycin. PMA (10ng/ml, Sigma-Aldrich) in combination with 200ng/ml or 1000ng/ml Ionomycin (sigma-Aldrich) were used to stimulate LN cells. The anit-CD3 (2μg/ml, clone 145-2C11, Biolegemd) antibody and anti-CD28 antibody (2μg/ml, clone 37.51, Biolegend) were also used to activate T cells. The designated concentrations (in text and figures) of Compound C (Sigma-Aldrich) and AICAR (Sigma-Aldrich) were used in our experiments. Each experiment contained a group with equal concentrations of DMSO as a control.

### Flow Cytometric analyses

Surface and intracellular staining were performed as what we previously described [38, 39]. Resting or activated T cells were acquired with a BD FACS Calibur. Flow cytometric analyses were performed with Flowjo (Tree Star). The following antibodies were used for surface staining, which included anti-CD4 (clone RM4-5), anti-CD8 (clone 53-6.7), anti-CD69 (clone H1.2F3), anti-CD25 (clone PC61), anti-CD71 (clone RI7217). For intracellular staining of cytokines, LN cells with different treatments were stimulated with PMA/Ionomycin and golgiplug (BD Bioscience) for 5 hours. The cells were collected and the cytokines were stained by anti-interferon-γ (IFNγ, clone XMG1.2), anti-IL-2 (clone JES6-5H4), or anti-TNFα (clone MP6-XT22) using the BD Cytofix/Cytoperm and Fixation/Permeabilization Solution kit.

### Analysis of T cell survival

T cell survival was analyzed by flow cytometric staining for Annexin V (BD Bioscience) and 7-AAD (BD Bioscience) at different conditions. For *in vitro* activation, cells from lymph nodes were activated with PMA/Ionomycin at indicated time points and collected for analysis. Annexin V and 7-AAD double negative population was considered as live T cells.

### Phosphorylation analysis by intracellular staining

For detection of phosphorylated proteins, cells from lymph nodes were cultured under different treatment conditions for the designated time points and were immediately fixed with Phosflow Lyse/Fix buffer (BD Biosciences) and permeabilized by Phosflow Perm buffer III (BD Biosciences). Cells were stained with the Alex488 conjugated antibody for p-S6^S235/236^ (clone D57.2.2E; Cell Signaling Technology), p-S6^S240/244^ (clone D68F8, Cell Signaling Technology) and p-Erk^T202/Y204^ (clone D13.14.4E Cell Signaling Technology). Cells were also stained with the antibody for p-AMPKα (Thr172) (clone 40H9, Cell Signaling Technology), p-ACC (Acetyl-CoA Carboxylase, Ser79) (clone D7D11, Cell Signaling Technology). FITC conjugated anti-Rabbit IgG (H+L) was used as a secondary antibody (Invitrogen, #65-6111).

### Western blotting

CD4^+^ T cells were isolated from lymph nodes with CD4^+^ T cells isolation kit (Stem cell). After pretreatment with Compound C or AICAR for 30 minutes, CD4^+^ T cells were stimulated with PMA/Ionomycin for another 20 minutes. Cells were lysed in buffers with protease and phosphorylation inhibitors. Protein concentrations were determined by BCA assay (Thermo Scientific). The antibodies of p-AMPK^T172^ (#4188), p-ACC^S79^ (#3661), p-S6^S235/236^ (#4803), p-S6^S240/244^ (#4803), p-ERK^T202/Y204^ (#4370), p-4EBP1^T37/46^ (#7547), p-AKT^S473^ (#4060), p-S6K^T389^ (#9205) were purchased from Cell Signaling Technology. β-actin was used as a loading control.

### ELISA

Mouse IL-2, IFNγ and TNF-α ELISA kit (Biolegend) was used to measure the level of IL-2, IFNγ and TNF-α in cultural supernatants according to manufacturer's protocol.

### Statistical analysis

Values shown in the figures represent means±SD. Unpaired, two-tailed Student's t test was performed for the comparison of results from different treatments. A p value of less than 0.05 is considered statistically significant.

## SUPPLEMENTARY MATERIAL FIGURES


