# Supplementary material for: AMPK-dependent and independent effects of AICAR and compound C on T-cell responses
Source: Oncotarget. 2016 May 10;7(23):33783–95. doi: 10.18632/oncotarget.9277 (PMC5085118; doi:10.18632/oncotarget.9277)
Supplement: Supplementary file 1 [file oncotarget-07-33783-s001.pdf]

AMPK-dependent and independent effects of AICAR and compound C on T-cell responses

Supplementary Material

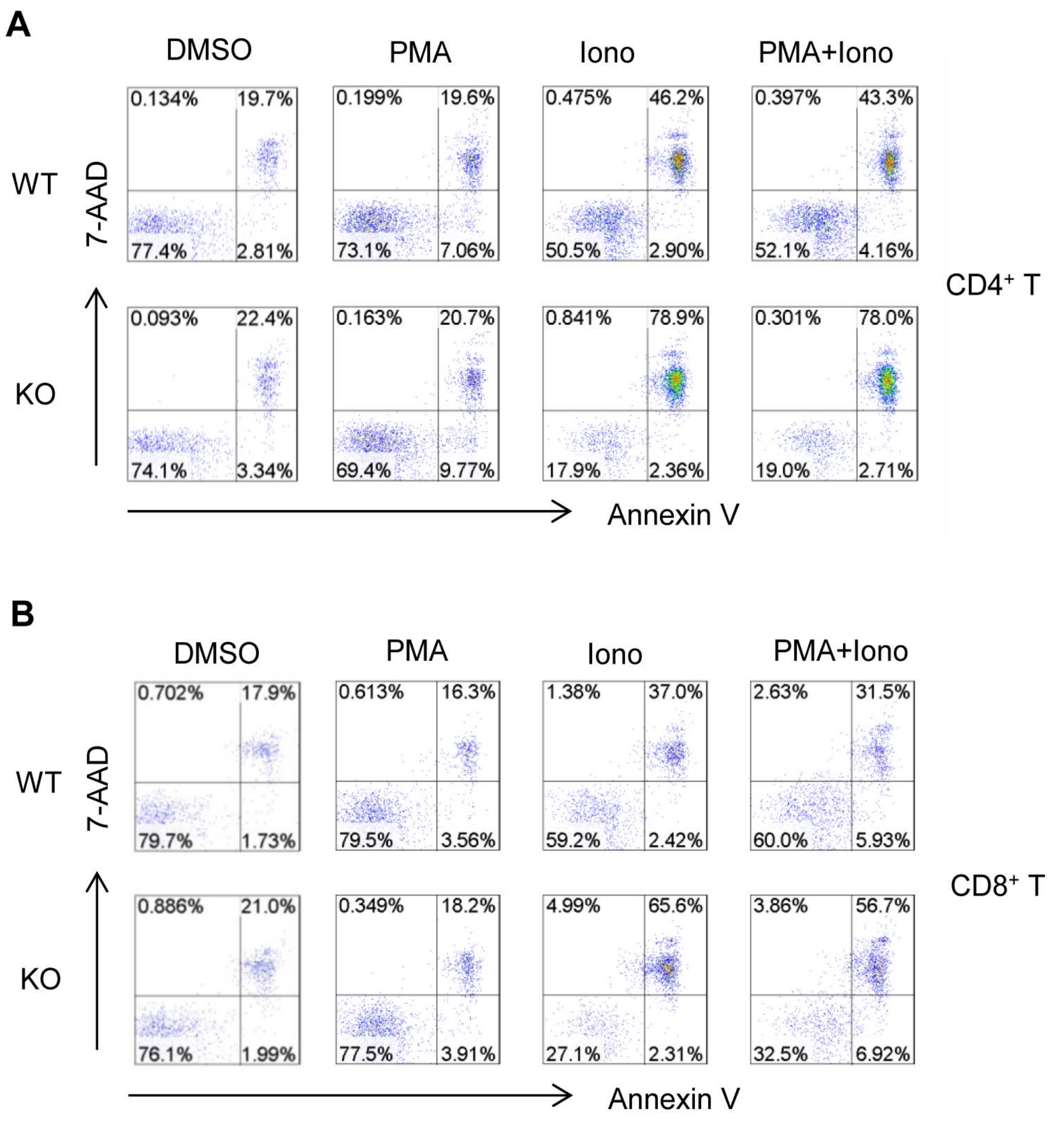

Supplementary Figure 1 Ionomycin, but not PMA, induces T cell death. Cells from lymph nodes of WT and KO mice were cultured with DMSO control, PMA (10ng/ml) alone, Ionomycin (1000ng/ml) alone, or PMA(10ng/ml) plus Ionomycin (1000ng/ml) for 6 hours. The survival of $CD4^+$ T cells (**A**) and $CD8^+$ T cells (**B**) was analyzed by flow cytometric staining with Anenexin V and 7-AAD. Data represent one of two independent experiments.

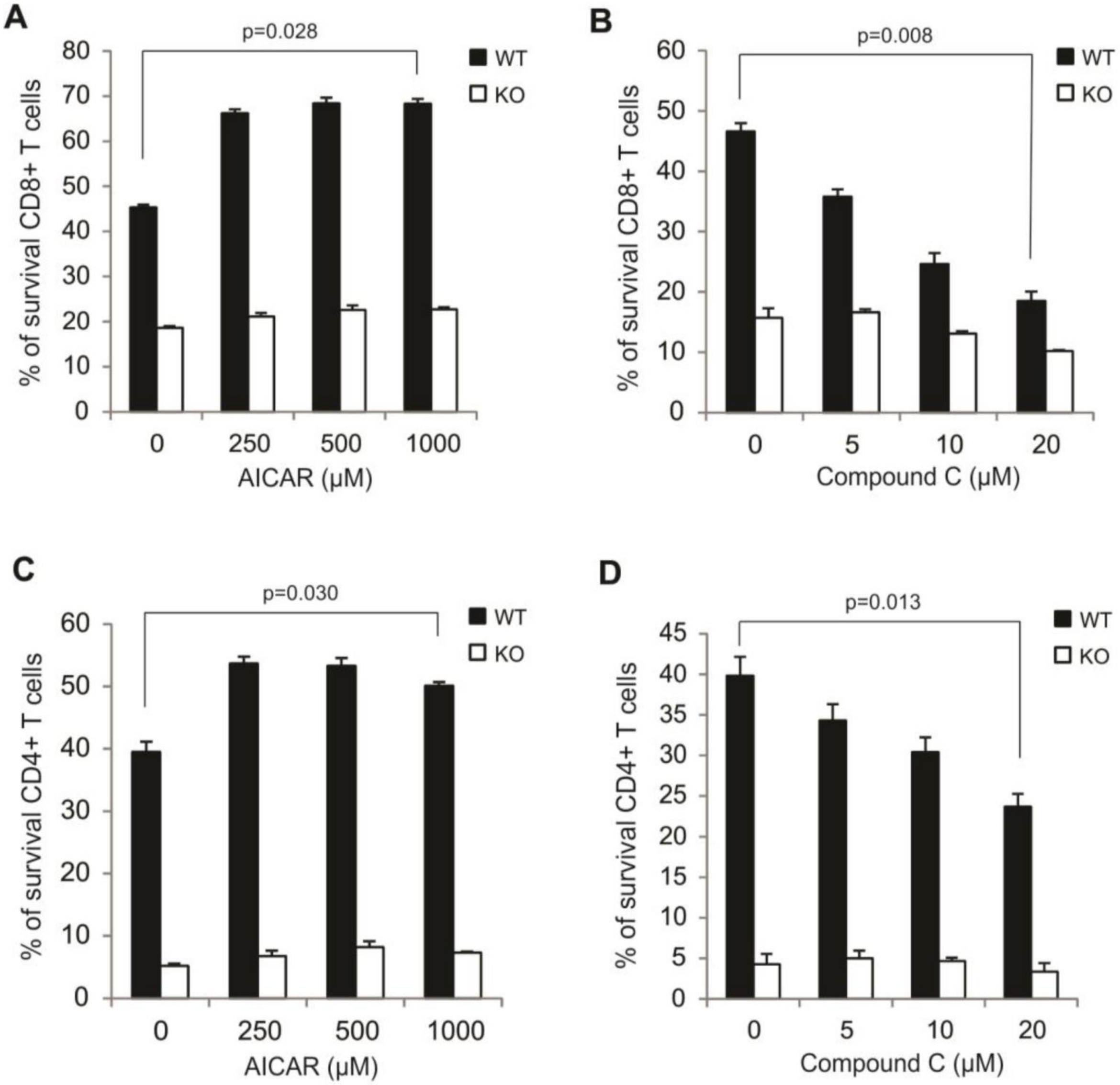

Supplementary Figure 2 AICAR inhibits, but Compound C promotes, Ca^{2+} signaling-induced T cell death in an AMPK-dependent manner. Cells from lymph nodes of WT and KO mice were pretreated with indicated concentrations of AICAR or Compound C for 30 minutes, and then stimulated with PMA (10ng/ml)/Ionomycin (1000ng/ml) for 12 hours. The survival of CD8$^+$ T cells and CD4$^+$ T cells was analyzed by flow cytometric staining with Annexin V and 7-AAD. **(A)** The survival of CD8$^+$ T cells with AICAR treatment. (B) The survival of CD8$^+$ T cells with compound C treatment. (C) The survival of CD4$^+$ T cells with AICAR treatment. (D) The survival of CD4$^+$ T cells with compound C treatment.

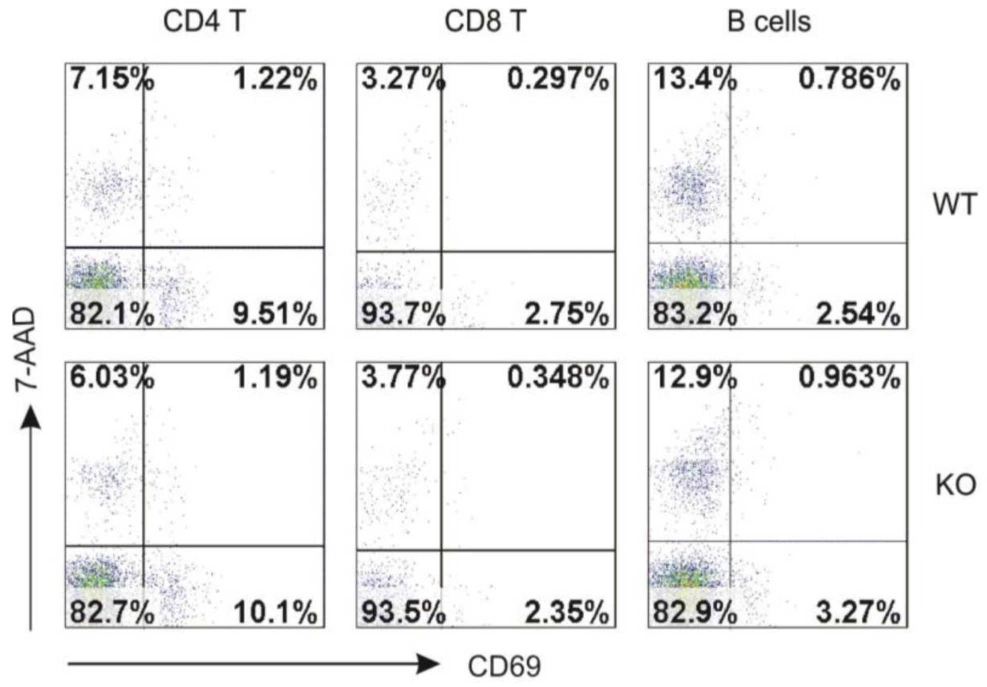

Supplementary Figure 3 CD69 is lowly expressed in lymphocytes. Cells from lymph nodes of WT and KO mice were analyzed for the expression of CD69 on $CD4^+$ T, $CD8^+$ T and B cells by flow cytometric staining with anti-CD69 antibody and 7-AAD staining.

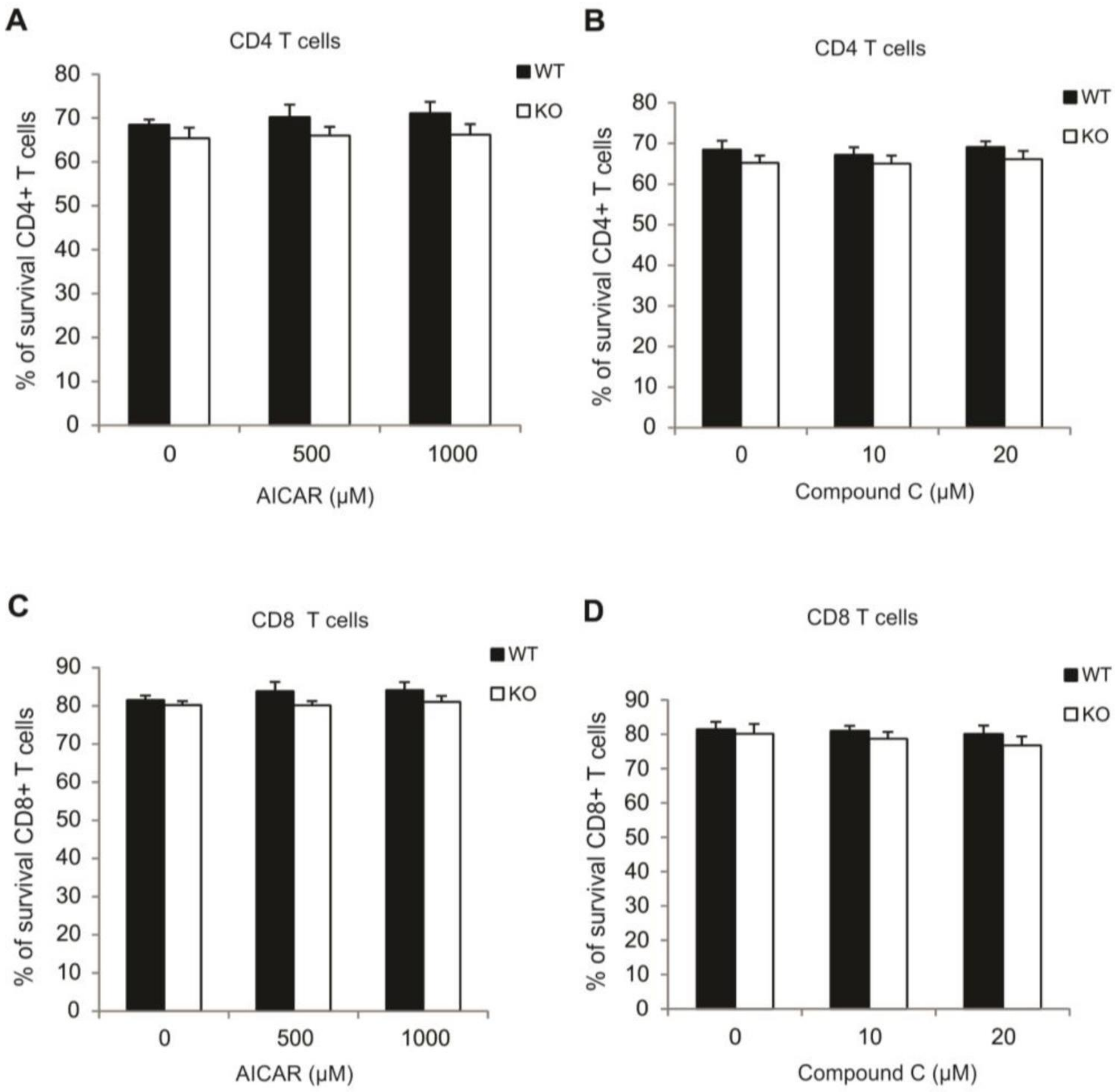

Supplementary Figure 4 Low-dose Ionomycin exerts minimal effects on T cell death with/without AICAR/Compound treatment between WT and KO mice LN cells from WT and KO mice were pretreated with indicated concentrations of AICAR or Compound C for 30 minutes, and then stimulated with Ionomycin (200ng/ml) for 6 hours. The survival of $CD4^+$ T cells and $CD8^+$ T cells treated with AICAR or Compound C, respectively, were analyzed with Annexin V and 7-AAD staining.

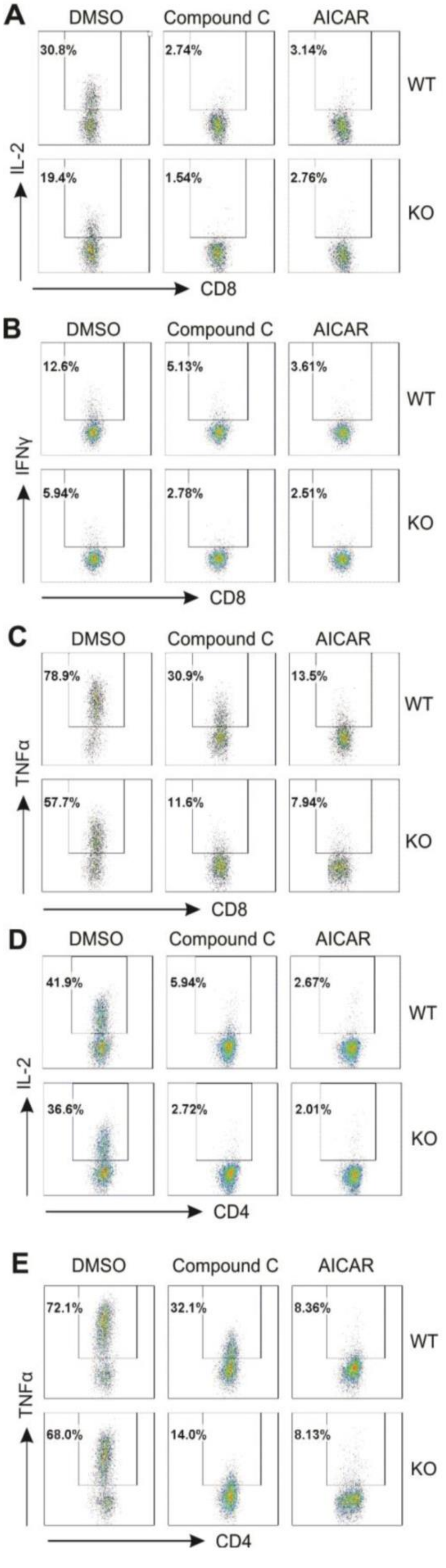

Supplementary Figure 5 AICAR and Compound C inhibit T cell cytokines production in an AMPK-independent manner. Cells from lymph nodes of WT and KO mice were pretreated with DMSO, Compound C (10µM) or AICAR (500µM) for 30 minutes, and then stimulated with PMA (10ng/ml)/Ionomycin (200ng/ml) and Golgiplug for 5 hours. Cells were collected and stained for cytokine production. **(A)** IL-2 production in $CD8^+$ T cells. **(B)** IFNγ production in $CD8^+$ T cells, **(C)** TNFα production in $CD8^+$ T cells. **(D)** IL-2 production in $CD4^+$ T cells. **(E)** TNFα production in $CD4^+$ T cells. Data represent one of at least two independent experiments.
